# Extra-pulmonary Tuberculosis among Tuberculosis Patients Visiting a Tertiary Care Centre: A Descriptive Cross-sectional Study

**DOI:** 10.31729/jnma.7231

**Published:** 2022-12-31

**Authors:** Damodar Paudel, Surendra Lal Shrestha

**Affiliations:** 1 Department of Internal Medicine, Nepal Police Hospital, Maharajgunj, Kathmandu, Nepal; 2 Department of Internal Medicine, National Academy of Medical Sciences, Mahaboudha, Kathmandu, Nepal

**Keywords:** *extrapulmonary*, *pulmonary*, *tuberculosis*

## Abstract

**Introduction:**

Tuberculosis outside of the lung is extrapulmonary tuberculosis. The diagnosis of extrapulmonary tuberculosis is not easy in a community setting even in district hospitals. Clinical symptoms and radiological diagnosis are effective for the diagnosis of pulmonary and extra-pulmonary cases. The aim of the study is to find out the prevalence of extra-pulmonary tuberculosis among tuberculosis patients visiting a tertiary care centre.

**Methods:**

This was a descriptive cross-sectional study conducted among the patient visiting a tertiary care centre from August 2019 to January 2020 after taking ethical approval from Institutional Review Committe (Reference number: 86) which observed different clinicoradiological and laboratory features in patients with extrapulmonary tuberculosis to address their respective accuracy and usability in measuring the burden of tuberculosis. The interview was taken for the symptoms, and then radiological and laboratory data were collected. Convenience sampling was used. Point estimate and 95% confidence interval were calculated.

**Results:**

Out of 60 patients with tuberculosis, 39 (65%) (62.83-67.17, 95% Confidence Interval) were diagnosed with extrapulmonary tuberculosis. Among them, 30 (76.9%) were with pleural effusions followed by lymphadenopathy in 9 (23.07%).

**Conclusions:**

The prevalence of extra-pulmonary tuberculosis was found to be higher as compared to the study done in similar settings.

## INTRODUCTION

Tuberculosis is one of the leading causes of death from a single infectious agent, ranking above HIV/AIDS.^1^ National data suggestive of 29% were bacteriologically or clinically diagnosed extrapulmonary TB.^2^ The most common extrapulmonary tuberculosis (TB) is lymph node followed by the intestine, miliary TB, bone/joints, meninges, skin, genital, larynx and TB in other sites like pericardium, breast, thyroid, salivary gland, soft tissue. Body fluid and biopsy are the hallmarks of the diagnosis.^3^

Extra efforts should be afforded in the community to not miss the extrapulmonary TB.^4^ Patients with strongly suspected tuberculosis disease and negative sputum microscopy or culture still get the diagnostic problem. It can be more difficult in asymptomatic patients, where its yield in tuberculosis detection rate is up to 21% of sputum-positive cases.^3,5^ Experienced clinicians can diagnose TB with radiological and common clinical symptoms.

The aim of the study was to find the prevalence of extrapulmonary tuberculosis among patients with tuberculosis in a tertiary care centre.

## METHODS

This is a descriptive cross-sectional study conducted among the tuberculosis confirmed adult patients visiting the Department of Medicine in National Academy of Medical Sciences (NAMS) in Bir hospital. The study was conducted from August 2019 to January 2020 after receiving ethical approval from the Institutional Review Committee (Reference number: 86). We have included the patients with following inclusion criteria: i) Patient with lymphadenopathy and positive fine needle aspiration cytology (FNAC), adenosine deaminase (ADA) significant to diagnose ascites and pleural effusion, ii) Clinical and radiological features suggestive of spine TB, or other suspected extra pulmonary TB signs, iii) Acid fast bacilli (AFB) positive or Chest X-ray (CXR) suggestive of TB (pleural effusion, fibrocavitary, infiltration, consolidation, fibrosis, collapse, miliary), iv) Positive cases (sputum AFB, GeneXpert), extra pulmonary, computed tomography (CT), magnetic resonance imaging (MRI) positive cases.

Any patient with concomitant cardio-vascular or chest disease that might affect his chest X-ray, and the patients below 15 years of age were excluded from the study.

The sample size was calculated by using the formula:


$$\begin{array}{ll} \text{n} & ={\text{Z}}^{2}\times \frac{\text{p}\times \text{q}}{{\text{e}}^{\text{2}}}\\ & =1.96^{2}\times \frac{0.48\times 0.52}{0.1^{2}}\\ & =66 \end{array}$$

Where,

n = required sample size
Z = 1.96 at 95% Confidence Interval (CI)
p = prevalence of patient with extrapulmonary tuberculosis taken from previous studies, 48.5%^5^
q = 1-p
e = margin of error, 10%

Convenience sampling technique was applied for tracing the study participant. Each of the TB patients was traced from the OPD or IPD of the hospital. After the proper informed consent face to face interview and the verification of the record file was used for collection of data. The predesigned performa was used as data collection tools.

Patients who are the possible case of TB with inclusion criteria presented to the emergency, outpatient or inpatient Department in Bir hospital was taken as participants of the study. Written informed consent was taken. Patient, who is unwilling to participate in the study, were not enrolled in the study. All patients' history detail and clinical examination was done. demography, clinical features laboratory parameter and outcome was recorded as per Performa. Blood, Sputum, Mantoux test investigations were routinely done without any financial burden to the patient. Findings of pleural fluid analysis done were noted. CT, X-ray finding was noted with the recent film and conformed to the radiologist report. Patients were seen till 1 week after ATT or until discharge from the hospital. All the data was analyzed and divided into two groups, according to our case definition in Pulmonary TB and extra pulmonary TB.

Data were entered and analysed with IBM SPSS Statistics version 23.0. Point estimate and 95% CI were calculated.

## RESULTS

Out of 60 patients included in our study, the prevalence of extrapulmonary tuberculosis is found to be 39 (65%) (62.83-67.17, 95% Confidence Interval). Twenty-five (64.10%) were males and 14 (35.90%) were females and male to female ratio is 1.8:1. Most of them were from the urban area i.e. 29 (74.36%) and 10 (25.64%) were from the rural areas. Most of them were farmers, followed by students and housewives, while there were least number of businessmen 1 (2.56%). Out of all, 4 (10.26%) were HIV serologic status (Table 1).

**Table 1:** Characteristics of the study population and the symptoms (n= 39).

Characteristics		n (%)
	16-40	26 (66.67)
Age (years)	40-50	4 (10.25)
	>50	9 (23.08)
Gender	Male	25 (64.10)
	Female	14 (35.90)
Domicile	Urban	29 (74.36)
	Rural	10 (25.64)
	Businessman	1 (2.56)
	Farmer	13 (33.33)
	Housewife	6 (15.38)
Occupation	Labor	3 (7.69)
	Prisoner	1 (2.56)
	Security guard	2 (5.13)
	Student	13 (33.33)
HIV status	Positive	4 (10.26)
	Fever	21 (53.85)
	Cough	20 (51.28)
	Chest Pain	9 (23.08)
Symptoms	Loss of appetite	16 (41.03)
	Weight loss	14 (35.90)
	Lymphadenopathy	10 (25.64)
	Hemoptysis	5 (12.82)

Among the extra pulmonary cases, 26 (66.67%) were pleural effusion followed by disseminated tuberculosis 5 (12.82%) and 3 (7.69%) abdominal TB (Table 2).

**Table 2:** Distribution of extrapulmonary tuberculosis (n= 39).

EPTB	n (%)
Pleural effusion	26 (66.67)
Dissiminated tuberculosis	5 (12.82)
Abdominal TB	3 (7.69)
TB meningitis	1 (2.56)
TB lymphadnitis	2 (5.12)
TB pericardial effusion	1 (2.56)
Miliary Tuberculosis	1 (2.56)
Total	39 (100)

Out of 39 extrapulmonary cases, 11 (28.20%) had loss of weight, 16 (41.02%) had loss of appetite and 21 (53.85%) had reported that they have evening rise of temperature (Table 3).

**Table 3:** Symptoms, age, locality and duration of stay in extra pulmonary tuberculosis (n= 39).

Symptoms	n (%)
Weight loss	11 (28.20)
Loss of appetite	16 (41.02)
Evening rise of temperature	21 (53.85)
Cough more than 2 week	20 (51.28)
Fatigability	9 (23.08)
Lymphadenopathy	9 (23.08)
Sputum positive	9 (23.08)
Mantoux test	2 (5.12)
Death	2 (5.12)
0-7 days	12 (30.77)
7-14 days	21 (53.84)
>14 days	6 (15.38)

Out of 39 extra-pulmonary tuberculosis patients included in this study, 9 (23.08%) patients had AFB positive sputum, GeneXpert sputum examination and diagnosis, 2 (5.12%) were sputum GeneXpert positive and 2 (5.12%) were positive for Mantoux test.Out of 13 pleural fluid positive extra pulmonary cases who had their body fluid tested for ADA, pleural fluid ADA positivity was 7 (53.84%). Among the 39 extrapulmonary cases, Ascitic was positive in 12.82% of patients. Amongst them, 66.6% were Ascitic fluid ADA positive (Table 4).

**Table 4:** Asitic and pleural fluid ADA (n = 39).

Pleural fluid	ADA		Total n
	Negative n (%)	Positive (%)	n (%)
Negative	5 (55.6)	2 (11.1)	7 (25.9)
Positive	4 (44.4)	16 (88.9)	20 (74.1)
**Asitic fluid**			
Negative	6 (66.7%)	17 (94.4)	23 (85.2)
Positive	3 (33.3%)	1 (5.6)	4 (14.8)

Patients were evaluated with imaging study i.e. Chest X-Ray, ultrasonography and CT scan. Out of all the 39 cases, 24 (61.53%) patient had pleural effusion followed by 3 (7.69%) fibrocavitation and consolidation respectively and 5 (12.82%) patients had normal radiological finding (Table 5).

**Table 5:** Radiological findings in extrapulmonary cases (n= 39).

Radiological diagnosis	n (%)
Normal	5 (12.82)
Fibrocavitary	3 (7.69)
Infiltration	1 (2.56)
Consolidation	3 (7.69)
Pleural effusion	24(61.53)
Lymphadenitis	1 (2.56)
Pericardial effusion	1 (2.56)
Multifocal lesion	1 (2.56)
Total	39 (100)

## DISCUSSION

Patient who was admitted in medical ward with the diagnosis of Tuberculosis (pulmonary and extrapulmonary)and the most of them (65%) were extra pulmonary tuberculosis. Pleural effusion was the most common finding in EPTB cases. Among the extra pulmonary cases 26 (66.6%) were Pleural effusion followed by disseminated tuberculosis 5 (12.8%) 3 (7.6%) abdominal TB. One of the retrospective cohort survey study done in Africa published less percentage of EPTB prevalence and the most common affected site was disseminated TB followed by pleura. This study is different than our study as it has more immune compromised HIV positive patient.^6^

In this study, young age, particularly 16-40 years were most infected with the extra pulmonary tuberculosis. This result showed the significantly different type of TB in different population of patients. The result of our study is similar to the study done in Manipal Medical College, however the author has distributed population in 25, 25-50 and more than 50 years.^7^ Male represented more 25 (64.10%) than female 14 (35.90%) in this study. Because of their biological and epidemiological causes, male are more prone to get tuberculosis, however, other studied reported as the bias as female has less access to health services. Most of the patients presented from the urban (74,%) area. Most of the patients in our study were of low socioeconomic background, most of them were farmers and students contained 66.6% of total extra pulmonary cases.

Among all the studied population, 4 (10.26%) were HIV positive, which is greater than published study from National tuberculosis center, Bhaktapur as it was 2.5%.^8^ Pleural effusion was the most common presentation of these patients.

On radiological imaging diagnosis, patient with the diagnosis of extra pulmonary TB had presented with pleural effusion which was most common unilateral and right sided, while there were less number, bilateral and left sided pleural effusion. Some of patients imaging study was normal. Similar study done in Manipal, Pokhara Nepal is different finding than in our study. They have enlisted the most common extra pulmonary tuberculosis is lymph nodes TB followed by a peritoneum and intestines.^5^

Mantoux test was performed in all patients, among them 5.12% were positive. Most of the extra pulmonary patents are low immune status. As Mantoux test is highly controversial test and low specific, it is difficult to consider as diagnostic test.^9^

In our study, 23.07% of patients were sputum positive for tuberculosis. The less percentage in sputum smears results may be due to the presence of cavitatory disease, technical error, less number of bacilli excreted per ml of sputum, and most of the cases were extra pulmonary as extra pulmonary cases are diagnosed by different fluid (ascitic fluid, pleural fluid, CSF ) and the bacteria are less in number, it is not easy to get them in sputum.^10^

Most of the patients who were admitted in ward with the diagnosis of extra pulmonary TB stay in ward for more than a week. In pleural fluid analysis, ADA was positive in 54.3% of the patient, while it was 66.6% positive in ascitic fluid analysis which is similar to some previous studies.^11^

## CONCLUSIONS

Extra-pulmonary tuberculosis is common in urban areas, mostly in low socioeconomic populations. The prevalence of extra-pulmonary tuberculosis is found to be higher as compared to the study done in similar settings.

## References

[1] El-Khushman H, Momani JA, Sharara AM, Haddad FH, Hijazi MA, Hamdan KA (2006). The pattern of active pulmonary tuberculosis in adults at King Hussein Medical Center, Jordan.. Saudi Med J..

[2] National Tuberculosis Center, Department of Health Services, Ministry of Health, Government of Nepal. (2018). National Tuberculosis Program Nepal [Internet]..

[3] Shrestha P, Khanal H, Dahal P, Dongol P (2018). Programmatic impact of implementing Genexpert Mtb/Rif assay for the detection of Mycobacterium Tuberculosis in respiratory specimens from pulmonary tuberculosis suspected patients in resource limited laboratory settings of Eastern Nepal.. Open Microbiol J..

[4] Ubaidi BAA (2018). The radiological diagnosis of pulmonary tuberculosis (TB) in primary Care.. J Fam Med Dis Prev..

[5] Sreeramareddy CT, Panduru KV, Verma SC, Joshi HS, Bates MN (2008). Comparison of pulmonary and extrapulmonary tuberculosis in Nepal- a hospital-based retrospective study.. BMC Infect Dis.

[6] Ohene SA, Bakker MI, Ojo J, Toonstra A, Awudi D, Klatser P (2019). Extra-pulmonary tuberculosis: a retrospective study of patients in Accra, Ghana.. PLoS One.

[7] Bhalla AS, Goyal A, Guleria R, Gupta AK (2015). Chest tuberculosis: Radiological review and imaging recommendations.. Indian J Radiol Imaging..

[8] Adhikari N, Bhattarai R, Basnet R, Tinkari BS, Gyawali BN, Joshi LR (2019). Prevalence of human immunodeficiency virus infection among tuberculosis patients in Nepal.. J Nepal Health Res Counc..

[9] Ballal SG (1995). International controversies in interpreting the mantoux test with special reference to saudi arabia.. J Family Community Med..

[10] Pourostadi M, Rashedi J, Mahdavi Poor B, Samadi Kafil H, Hariri-Akbari M, Asgharzadeh M (2018). Frequency of smear-negative tuberculosis in Northwest Iran.. Iran J Med Sci..

[11] Christensen AS, Roed C, Andersen PH, Andersen AB, Obel N (2014). Long-term mortality in patients with pulmonary and extrapulmonary tuberculosis: a Danish nationwide cohort study.. Clin Epidemiol..

